# The comparison of inflammation markers in patients with non-valvular atrial fibrillation using warfarin and switched to apixaban

**DOI:** 10.12669/pjms.42.1.13059

**Published:** 2026-01

**Authors:** Omer Ozkan Duman, Erkan Alpaslan

**Affiliations:** 1 Omer Ozkan Duman, MD, Department of Cardiology, Izmir Demokrasi University, Goztepe, Izmir, Turkey; 2 Erkan Alpaslan Assistant Professor, Department of Cardiology, Izmir Demokrasi University, Goztepe, Izmir, Turkey

**Keywords:** Atrial fibrillation, Inflammation marker, Warfarin, Apixaban

## Abstract

**Objective:**

To compare the changes in levels of inflammation markers in the patient with non-valvular atrial fibrillation (NVAF) who were on Warfarin and then switched to Apixaban.

**Methodology:**

The files of 149 consecutive patients, who had NVAF, who were previously using Warfarin, but for various reasons switched to Apixaban were screened retrospectively. Forty-one patients were excluded from the study. Last coagulation parameters were used for analysis. The evaluation of hemogram parameters in patients undergoing treatment with Warfarin, subsequently transitioning to Apixaban, revealed significant insights into various hematological indices. Notably, the Neutrophil/Lymphocyte Ratio, Platelet/Lymphocyte Ratio, the Systemic Immune-Inflammation Index, Systemic Inflammation Response Index (SIRI), the aggregate index of systemic inflammation and Monocyte/Lymphocyte Ratio were meticulously assessed and compared between the groups.

**Results:**

The study involved 108 individuals with NVAF. The average lymphocyte level of the patients on Apixaban therapy was statistically significantly higher than on Warfarin therapy (p<0.001). Also, The Neutrophils (p<0.001), Monocytes (p=0.008), and Platelets (p<0.001) were significantly lower in patients treated with Apixaban compared to those on Warfarin. When the systemic inflammation indices of the patients were compared, the averages of the patients’ NLR, PLR, MLR, SII, SIRI, and AISI indices were statistically and significantly lower than time on Warfarin therapy.

**Conclusion:**

Current study results suggested that Apixaban has more effective anti-inflammatory potential compared to Warfarin. The study implicates new perspectives on the anti-inflammatory effects of oral anticoagulants used in the treatment of NVAF.

## INTRODUCTION

In clinical practice, Atrial Fibrillation (AF) is the most common arrhythmia associated with increased morbidity and mortality as it is associated with increased risk of ischemic stroke and systemic embolism.^1^,^2^ Inflammation plays important roles in the pathogenesis of non-valvular AF (NVAF). Previous studies have reported that there is a close relationship between the prevalence and prognosis of NVAF and the levels of inflammation markers.^3^,^4^ The most commonly used oral anticoagulant to prevent stroke and systemic embolism in NVAF was Warfarin, a vitamin K antagonist.^5^

However, the use of Warfarin requires frequent monitoring and dose adjustment because of its narrow therapeutic range and interactions with various drugs and foods. Novel oral anticoagulants such as Dabigatran, Apixaban, Rivaroxaban and Edoxaban, which directly inhibit Factor Xa (FXa), have been developed to overcome the difficulties with warfarin therapy. Direct oral anticoagulants employed in preventing stroke and systemic embolism in the treatment of NVAF have been shown to be at least as effective as Warfarin. There is no need for monitoring, and more importantly, they have a lower bleeding risk.^6^-^9^ Factor Xa plays important roles in the activation of the inflammatory response.^10^ Anti-FXa drugs might have additional potential advantage like anti-inflammatory effects.^11^

Elevated C-Reactive Protein (CRP) and Interleukin-6 (IL-6) are independent stroke risk factors.^12^,^13^ As well as CRP and IL-6, inflammation-associated indices derived from Neutrophil, Monocyte, Lymphocyte, and Platelet counts obtained in complete blood count are used to evaluate inflammatory activity. Among these, Neutrophil/Lymphocyte (NLR) and Platelet/Lymphocyte (PLR) Ratios have been shown to have significant effects on predicting the prognosis of cardiovascular diseases (CVD) and CV mortality.^14^,^15^ Three recent inflammatory markers (Systemic Immune Inflammation Index (SII), Systemic Inflammatory Response Index (SIRI), and Systemic Inflammatory Aggregate Index (AISI)) have provided additional data in CVD risk evaluation.^16^,^17^ The present study aimed to compare the changes in the levels of inflammation markers in the patient who were on Warfarin and then switched to Apixaban.

## METHODOLOGY

All patients who presented to our clinic during the specified study period, initiated Warfarin therapy, and were subsequently switched to Apixaban treatment were included in the study. The dose of apixaban was adjusted according to age and body weight, and administered as either 2.5 mg or 5 mg twice Daily.

### Ethical Approval:

The study was approved by the Non-Interventional Research Ethics Committee of Buca Seyfi Demirsoy Training and Research Hospital (Approval No. 2024/233, Dated: 31/01/2024) and conducted in accordance with the Declaration of Helsinki. Informed consent was waived due to the retrospective nature.

### Inclusion Criteria:

We retrospectively screened the files of 149 consecutive patients with non-valvular atrial fibrillation, who presented to the Cardiology Clinic between June 2023 to January 2024, and were previously on Warfarin therapy, but for various reasons (e.g., not being in the effective therapeutic range [n=13, 31.7%], inability to attend follow-ups [n=16, 39%], food/drug interactions [n=4, 9.7%], patient preference [n=8 19.5%], etc.), were switched to Apixaban in the present study. The demographic data and lab results from patients were extracted from hospital archives. We used the data which was obtained a uniform laboratory protocol for blood analyses. Blood samples were collected at the third month after the initiation of treatment were recorded for both groups. In the Warfarin phase, hemogram and coagulation parameters were analyzed at the three-month follow-up before the switching to the apixaban. In the Apixaban phase, hemogram parameters were recorded from samples taken at the three-month follow-up after starting Apixaban therapy. Inflammatory markers were evaluated only at three months post-transition. Thus, the follow-up duration for this study was standardized to three months post-transition for all patients involved.

### Exclusion Criteria:

Participants were excluded if they had a history of malignancies, thyroid or autoimmune disorders, chronic kidney disease, acute infections, cancer, rheumatological diseases, transient inflammatory conditions, or borderline health conditions were excluded. Those who used antiplatelet agents, NSAIDs and other potential medications that may affect the patient’s hemogram in the last four weeks were also excluded. Additionally, individuals with alcohol use disorder, severe dyslipidemia, bleeding disorders, liver disease, pregnancy, puerperium, or breast feeding were excluded. (n=41 patients) from the study. A total of 108 patients remained eligible for inclusion in the study cohort.

The G-Power version 3.1.9.4 software was employed to determine the required sample size, taking into account the significance level and effect size of the hypothesis. In pursuit of the outlined objective, a comprehensive analysis will be undertaken to discern the variances in terminal p values across the designated groups. Consequently, the requisite minimum sample size for each group has been derived, maintaining a Type-I error rate set at α=0.05, a statistical power of 0.80, and an effect size quantified at d=0.50, culminating in a total of 102 participants, with 51 individuals allocated to each group. Furthermore, in anticipation of potential discrepancies arising from incomplete data within patient records, a strategic augmentation of the sample size by an additional 10% has been proposed, thereby incorporating 10 supplementary individuals into the cohort. Such a meticulous approach seeks to bolster the robustness of the findings while ensuring the integrity of the data collection process.

Last laboratory and coagulation parameters were used for analysis. The evaluation of hemogram parameters in patients undergoing treatment with Warfarin, subsequently transitioning to Apixaban, revealed significant insights into various hematological indices. Notably, NLR, PLR, and Monocyte/Lymphocyte Ratio (MLR) were meticulously assessed. Additionally, the Systemic Immune-Inflammation Index (SII), calculated as Platelet multiplied by Neutrophil divided by Lymphocyte count, along with the Systemic Inflammation Response Index (SIRI), derived from the product of Neutrophil and Monocyte counts divided by Lymphocytes, were also determined. Furthermore, the aggregate index of systemic inflammation (AISI), defined as the product of Neutrophil, Monocyte, and Platelet counts divided by Lymphocyte counts, was computed.

### Statistical Analyzes:

The data were analyzed with the Statistical Package for the Social Sciences 26.0 (SPSS 26.0) software. Numbers (n), percentages (%), mean values (*χ̅*), Standard Deviation values (±SD), median, minimum (Min.), and maximum (Max.) values were used to analyze the data. The normality of the distribution was checked with the Kolmogorov-Smirnov Test in the study. The Mann-Whitney U Test was used for non-parametric distributions to compare the means of two independent groups. The Paired Samples t-test was used for parametric distributions and the Wilcoxon Signed Ranks test was used for non-parametric distributions in comparing the means of two dependent groups. It was considered significant when p< 0.05.

## RESULTS

A total of 108 patients who were diagnosed with non-valvular AF were included in the present study. The basic characteristics of the patients included are given in Table-I and the descriptive characteristics of the patients and their comparisons according to their INR levels are given in Table-II. About 58.3% of the study group was female. The mean age of the patients was 72.6±7.2 years, and 57.4% were younger than 75 years old. The average INR level of the group while using Warfarin was 2.65±0.96. No statistically significant differences were detected between the INR levels of the participants according to their gender and age groups (p>0.05). No dose or medication changes were observed during the patients’ treatment follow-up.

**Table-I:** Basic characteristics of the patients.

Characteristics	Switching from Warfarin to Apixaban (n=108)
Age (y), Mean ± SD	72.6±7.2
***Sex, n (%)***	
Female	63(58.3%)
Male	45(41.7%)
***Smoking, n (%)***	
Current	12(11.1%)
Ex	22(20.3%)
Non	74(68.6%)
***Comorbidities, n (%)***	
Stroke/TIA	0 (0%)
Ischemic heart disease	21(19.4%)
Diabetes Mellitus	15(13.8%)
Hypertension	48(44.4%)
Hyperlipidemia	29(26.8%)
Venous Thromboembolism	0 (0%)
Chronic kidney disease/Creatinine(mg/dl)	0 (0%) /0.93±0.35
Chronic liver disease	0 (0%)

**Table-II:** The descriptive characteristics of the patients and comparison of these characteristics according to INR levels.

Characteristics	n(%)	INR, Mean ± SD	p-value
*Sex*			
Female	63(58.3%)	2.56±0.91	0.376
Male	45(41.7%)	2.77±1.03	
*Age*			
<75	62(57.4%)	2.61±0.87	0.913
>75	46(42.6%)	2.69±1.08	

INR: International Normalized Ratio.

A comparison of complete blood count findings in patients who used Warfarin for at least two months and were switched to Apixaban is given in Table-III. The average lymphocyte level of the patients on Apixaban was significantly higher the period on Warfarin therapy (p<0.001). Also, Neutrophil (p<0.001), Monocyte (p=0.008), and Platelet (p<0.001) levels were statistically significantly lower compared to the period when Warfarin was used.

**Table-III:** The comparison of complete blood count findings of patients using Warfarin and switching to Apixaban because of non-valvular AF.

Parameters	Warfarin	Apixaban	p-value
Neutrophils,× 10^9^/L	4.97±1.15	4.52±1.10	<0.001
Lymphocytes,×10^9^/L	1.96±0.56	2.30±0.72	<0.001
Monocytes,×10^9^/L	0.61±0.19	0.57±0.17	0.008
Platelet,× 10^9^/L	256.83±64.19	236.48±62.50	<0.001

When the systemic inflammation indices of the patients were compared, the averages of the patients’ NLR, PLR, MLR, SII, SIRI, and AISI indices on Apixaban therapy were statistically and significantly lower than in the period on Warfarin (Table-IV).

**Table-IV:** The comparison of the systemic inflammation indices of patients using Warfarin and switching to Apixaban because of non-valvular AF.

Parameters	Warfarin	Apixaban	p-value
NLR	2.77±1.15	2.16±0.88	<0.001
PLR	142.16±55.30	114.14±52.53	<0.001
MLR	0.34±0.17	0.27±0.11	<0.001
SII	695.41±329.47	506.27±259.99	<0.001
SIRI	1.69±0.95	1.21±0.59	<0.001
AISI	433.88±307.73	288.03±170.16	<0.001

NLR, neutrophil-to-lymphocyte ratio; PLR, platelet-to-lymphocyte ratio;

MLR, monocyte-to- lymphocyte ratio; SII, systemic immune-inflammation index;

SIRI, systemic inflammation response index; AISI, aggregate index of systemic inflammation.

## DISCUSSION

The primary findings of our study showed that iInflammation indices derived from complete blood counts revealed significant reductions in patients who switched from Warfarin to Apixaban . Systemic inflammation indices, including NLR, PLR, MLR, SII, SIRI, and AISI, were all significantly lower during Apixaban therapy compared Warfarin therapy. Apixaban exhibits a more potent anti-inflammatory effect than Warfarin in NVAF patients, as evidenced by the favorable shifts in hemogram parameters and derived indices. To our knowledge, this is the first study to directly compare complete blood count-based inflammation markers between Warfarin and Apixaban in the same patient cohort using a within-patient switch design.

Increasing evidence supports the roles of inflammation in the pathophysiology of NVAF, and the inflammatory process is considered to be a potential therapeutic target.^18^,^19^ Some case-control studies have reported that inflammatory markers (e.g., CRP, IL-1, IL-6, TNF), as well as Neutrophils and Lymphocytes, are elevated in patients with NVAF when compared to those in sinus rhythm.^20^ The mechanisms underlying the inflammatory process and dynamic changes in patients who develop AF might vary in different clinical scenarios and specific and personalized anti-inflammatory strategies targeting specific inflammatory cascades must be developed.

However, so far, no drugs have been designed to specifically target the inflammatory pathways in patients with NVAF. Studies were conducted with various drugs that had anti-inflammatory effects to reduce the development or recurrences of NVAF. In a previous study that was conducted with Statins, which have pleiotropic effects as well as cholesterol-lowering effects, reported that Rosuvastatin reduced the risk of NVAF compared to placebo.^21^ However, meta-analyses suggest that Statins might not be primarily effective in preventing NVAF.^22^,^23^ Statins did not prevent NVAF recurrence following cardioversion and catheter ablation.^21^,^24^ Corticosteroids, another drug group with anti-inflammatory effects, reduced postoperative AF (POAF) development and NVAF recurrence following catheter ablation.^25^,^26^

In a previous study that investigated the effects of Colchicine, it was reported that Colchicine administered postoperatively prevented POAF.^27^ Nevertheless, fixed-dose Colchicine that was started preoperatively failed to prevent POAF in another study.^28^ As a result, the net clinical effects of anti-inflammatory drugs in NVAF are limited. Factor Xa inhibitors, Apixaban and Rivaroxaban have anti-inflammatory characteristics as well as their anticoagulant effects.^11^ These data are compatible with the findings obtained in the present study. The present study showed that there was a decrease in inflammatory markers after switching to Apixaban. The findings of the present study were consistent with the data supporting that the anti-inflammatory effects of factor Xa inhibitors. While related studies, such as Martins et al.^29^, demonstrated lower levels of inflammatory cytokines (e.g., IL-6, TNF-α) in NVAF patients treated with rivaroxaban compared to warfarin, their cross-sectional design compared separate patient cohorts, potentially introducing confounding variables like baseline differences in disease severity or comorbidities. In contrast, the current study employs a within-patient switch design, allowing for direct comparison of inflammation markers before and after transitioning to apixaban, thereby minimizing inter-individual variability and strengthening causal inference regarding apixaban’s anti-inflammatory superiority.

Similarly, Katoh et al.^11^ reported reduced CRP and IL-6 levels with FXa inhibitors (including rivaroxaban and apixaban) versus warfarin in Japanese AF patients, but their focus was on broader FXa class effects without isolating apixaban or using a longitudinal switch approach. They conducted an observational study on patients without a direct comparison in the same population who switched drug therapy, relying on past trials for context. In contrast, in our study, we meticulously assess the inflammatory markers in the same cohort of patients both during their warfarin regimen and following their transition to apixaban. Such a design effectively mitigates confounding variables, thereby providing robust evidence to substantiate the superior efficacy of apixaban over warfarin. Moreover, while Katoh et al. concentrated on plasma biomarkers such as IL-6, pentraxin 3 and D-dimer, our study emphasizes the utilization of complete blood count-derived indices, specifically NLR and SII. This methodology not only streamlines the process but also aligns with standard clinical practices, rendering it a more accessible and cost-effective means of evaluating inflammation in everyday healthcare settings.

A potential mechanism underlying these observations may involve FXa inhibition reducing protease-activated receptor (PAR) signaling, particularly PAR-1 and PAR-2, which otherwise promotes proinflammatory cytokine release and endothelial activation in atrial tissue.^10^ Inflammation markers were shown to be a risk factor indicating the development of new-onset NVAF following Coronary Artery Bypass Graft surgery.^30^,^31^ Moreover, elevation of these markers was found to be associated with an increased risk of thromboembolic stroke in patients with NVAF.^32^

Beyond PAR signaling, Factor Xa is essential in hemostasis, promoting coagulation and interacting with monocytes to upregulate proinflammatory cytokines like IL-6 and TNF-α. This interaction exacerbates inflammation through increased monocyte adhesion and migration. Studies indicate that a selective Factor Xa inhibitor, may reduce monocyte activation and inflammation.^33^,^34^ Our research shows a marked decrease in monocyte populations and alterations in MLR and SII, highlighting apixaban’s anti-inflammatory effects.

The neutrophil-to-lymphocyte ratio serves as an important biomarker in NVAF, with high levels signifying an elevated risk of thromboembolic events.^35^ Monitoring NLR is essential. Recent evidence indicates that NLR decreases significantly after initiating apixaban therapy, suggesting this anticoagulant may reduce thromboembolic risks.^36^ Apixaban’s anti-inflammatory properties contribute to better outcomes seen in trials like ARISTOTLE, where it markedly cut the risk of stroke and systemic embolism compared to warfarin.^37^ By reducing inflammatory mediators, apixaban improves anticoagulation and adjusts inflammatory pathways, potentially enhancing treatment for Atrial Fibrillation patients. Its role underscores the importance of addressing inflammation in NVAF management.

Recent studies show a link between inflammatory markers and NVAF recurrence rates. Indices such as NLR, SII, and SIRI are tied to increased NVAF recurrence, especially post-cryoablation.^38^ Our investigation suggests that the initiation of apixaban therapy results in a reduction of inflammatory markers, which couldreduce the likelihood of atrial fibrillation recurrence and offer protective benefits by modulating inflammatory pathways. This finding is significant given the association between elevated SII and an increased risk of stroke and mortality.

Our findings could significantly reshape anticoagulant selection for patients with NVAF. Evidence increasingly favors direct oral anticoagulants like Apixaban over traditional vitamin K antagonists such as Warfarin. Clinicians navigate a complex interplay of factors-efficacy, safety, convenience, and cost-when determining the best therapeutic approach. The emerging anti-inflammatory advantages may play a critical role, especially for patients with high baseline inflammatory markers or increased thromboembolic risk. These insights could drive revisions in clinical guidelines or lead to personalized treatment strategies considering the inflammatory context in anticoagulation therapy.

It is clear that the predictability of NVAF will increase when the underlying pathophysiology of NVAF becomes more understood. Inflammatory indices based on simple and inexpensive complete blood counts were used in the present study. As a result of our study, it was found that inflammation activity decreased in the group of patients with NVAF who were switched from Warfarin to Apixaban. This effect of Apixaban on blood components (when compared to Warfarin) in patients with NVAF might be associated with its greater anti-inflammatory potential. This result might have given us an additional reason to prefer Factor Xa inhibitors rather than Warfarin in the treatment of Non-valvular AF.

### Limitations:

The results of the present study must be evaluated considering some limitations. First, the study was conducted in a single-center and in retrospective design which introduces confounding variables, and limits causal inferences. Second, the modest sample size and single-center nature limit broader applicability. Third, the quantity of participants in the research was quite limited, and the findings cannot provide a projection for patients using oral anticoagulants for indications other than NVAF. Forth, the sample size and cross-sectional design of the study on the potential anti-inflammatory effects of Apixaban and its clinical implications may be inadequate. Fifth, in the realm of clinical research, the absence of a non-switch control group-such as patients who continue on Warfarin-hinders our ability to conclusively rule out phenomena like regression to the mean or time-dependent effects that are not intrinsically linked to the treatment switch. Sixth, the studied cohort comprised entirely of Turkish individuals, all hailing from Izmir, Turkey, predominantly Turkish ethnic background. This homogenity may impose constraints on the generalizability of findings, particularly to diverse ethnic populations where genetic variations in coagulation and inflammatory pathways could significantly alter therapeutic responses. Seventh, the omission of direct inflammatory biomarkers like C-reactive protein and interleukin-6, alongside measured blood indices could enhance the understanding of the inflammatory context. For this reason, Future studies on cytokines like IL-6 and CRP, alongside hematological factors, may improve understanding of how anticoagulant treatments relate to inflammatory markers in atrial fibrillation management.

## CONCLUSION

Many previous studies have showed that inflammation plays roles in the pathophysiology of NVAF and the anti-inflammatory effects of Factor Xa inhibitors. In the present study, the changes in inflammatory markers after switching from Warfarin to Apixaban were uncovered in the same patient group. This result showed the possibility that Apixaban has a more effective anti-inflammatory potential when compared to Warfarin. However, this result must be confirmed with larger prospective studies with more participants. The results of the present study showed the need for new perspectives on the anti-inflammatory effects of oral anticoagulants used in the treatment of NVAF.

### Authors’ Contribution:

OD: Study conception and design, data collection and writing manuscript. Responsible and accountable for the accuracy and integrity of the work.

EA: Analysis and interpretation of results and writing manuscript.

All authors have read and approved the final version of the manuscript.
